# Intraoperative Magnetic Resonance Imaging Assisted Endoscopic Endonasal Resection of Clival Chordomas

**DOI:** 10.3389/fonc.2021.733088

**Published:** 2022-01-10

**Authors:** Beste Gulsuna, Burak Karaaslan, Memduh Kaymaz, Hakan Emmez, Emetullah Cindil, Muammer Melih Sahin, Emrah Celtikci

**Affiliations:** ^1^ Department of Neurosurgery, Gazi University Faculty of Medicine, Ankara, Turkey; ^2^ Department of Radiology, Gazi University Faculty of Medicine, Ankara, Turkey; ^3^ Department of Otorhinolaryngology, Gazi University Faculty of Medicine, Ankara, Turkey

**Keywords:** chordoma, intraoperative magnetic resonance imaging, skull base, IO-MRI, endoscopic

## Abstract

**Background:**

Cranial base chordomas are typically indolent and usually appear as encapsulated tumors. They slowly grow by infiltrating the bone, along with the lines of least resistance. Due to its relationship with important neurovascular structures, skull base chordoma surgery is challenging.

**Objective:**

The usefulness of intraoperative magnetic resonance imaging (IO-MRI) in achieving the goal of surgery, is evaluated in this study.

**Methods:**

Between March 2018 and March 2020, 42 patients were operated on for resection of skull base chordomas in our institution. All of them were operated on under IO-MRI. Patients were analyzed retrospectively for identifying common residue locations, complications and early post-operative outcomes.

**Results:**

In 22 patients (52,4%) gross total resection was achieved according to the final IO-MRI. In 20 patients (47,6%) complete tumor removal was not possible because of extension to the petrous bone (8 patients), pontocerebellar angle (6 patients), prepontine cistern (4 patients), temporobasal (1 patient), cervical axis (1 patient). In 13 patients, the surgery was continued after the first IO-MRI control was performed, which showed a resectable residual tumor. 7 of these patients achieved total resection according to the second IO-MRI, in the other 6 patients all efforts were made to ensure maximal resection of the tumor as much as possible without morbidity. Repeated IO-MRI helped achieve gross total resection in 7 patients (53.8%).

**Conclusions:**

Our study proves that the use of IO-MRI is a safe method that provides the opportunity to show the degree of resection in skull base chordomas and to evaluate the volume and location of the residual tumor intraoperatively. Hence IO-MRI can improve the life expectancy of patients because it provides an opportunity for both gross total resection and maximal safe resection in cases where total resection is not possible.

## Introduction

Chordoma is an uncommon, locally aggressive tumor originating from remnants of the primitive notochordal tissue along the cranial-spinal axis, which accounts 1% to 4% of all bone malignancies and constitutes 0.1% to 0.2% of all primary intracranial neoplasms (1–4). Approximately 35% of chordomas occur in the skull base predominantly in the clivus, cavernous sinus, and petrous apex (1, 3, 4). Cranial base chordomas are often indolent lesions because of their typically slow growth pattern (1–3, 5). They progress by infiltrating the low-resistance areas of the bone so cause the local destruction of the anatomic markers (1–3, 5). They may also envelop or compress the dura and other neurovascular structures adjacent to the tumor site, thereby causing brain stem or cranial nerve symptoms (1, 4).

There are various arguments regarding the optimal therapy for skull base chordomas (5–7). In most cases, maximal resection followed by adjuvant radiation therapy for the residual or recurrent tumor is recommended (5, 6, 8). However, due to the rarity of the disease, a standard treatment guideline has not been established yet (4, 5, 7, 9). Also, some studies showed that some tumor biological features, such as histological factors, Ki 67 labeling index, chromosomal abnormalities may affect survival (10, 11). Chordomas show varying degrees of radioresistance and a tendency to recurrence, so total tumor resection prolongs progression-free survival (1, 4–8, 10).

The development of intraoperative imaging and physiological monitoring methods in the last two decades has both supported the understanding of surgical anatomy and contributed to the safe removal of even the most complicated skull base lesions by surgeons (12). Surgical navigation systems based on preoperative imaging methods have been developed and are used in current practice, but the accuracy rate of these systems decreases significantly with surgical intervention, tumor resection, and CSF drainage (12, 13).

Intraoperative imaging methods are very useful and effective in the surgery of skull base lesions, due to the complex anatomical structure of this region, the destruction of some landmarks by the lesion, and the loss of three-dimensional relationship with important neurovascular structures (12, 14). There are a few analyses involving small patient groups for the value of IO-MRI in resection of skull base chordoma (4, 15). In this article, the usefulness of IO-MRI in achieving the goal of surgery is discussed. We present our experience with 42 patients with skull base chordomas, which is the first case series of skull base chordomas operated with a fully endonasal endoscopic approach combined with neuronavigation system and under IO-MRI reported in the literature.

## Materials and Methods

The study and usage of IO-MRI was approved by the Gazi University Clinical Research Ethics Committee with the approval number 2020-596. All patients were informed about the technique and gave their signed consent to IO-MRI during tumor resection and to the data being used for research purposes.

We performed a retrospective analysis of patients operated on for resection of skull base chordomas from March 2018 to March 2020. Admission and discharge notes, hospital records, surgery notes, preoperative radiological imaging, IO-MRI and follow-up control imaging were used as data sources. The patient population, preoperative clinical features, radiologic images, tumor extension, intraoperative images, location of the residual tumor, and postoperative complications were analyzed.

Before surgery, all patients were evaluated radiographically, MRI with and without contrast, tumor relation with the critical neurovascular structures is defined. High-resolution computed tomography (CT) with bone window was performed in all patients to evaluate the bony structures and extent of bone invasion.

All surgeries were performed under general anesthesia with orotracheal intubation. After the intubation, the patient was placed in the lateral decubitus position, the iliac crest was palpated to estimate the position of the L4-5 interspace. After a 14-gauge Tuohy needle was inserted at the midline through the interspinous space to the thecal sac, a drainage catheter was placed. Following the catheterization, the patient has placed supine position and the neuronavigation system (StealthStation^®^ Medtronic^®^ Inc, Minneapolis, USA) was set up. Rigid endoscopes (Karl Storz Endoskope^®^, Tuttlingen, Germany) 4 mm diameter, 18 cm length, and equipped with 0°, 30°, 45°, and 70° lenses, according to the different steps of the surgery, were used.

For use at closure, the Hadad-Bassagasteguy flap which is a pedicled nasoseptal flap that is vascularized by the sphenopalatine artery was prepared. This flap is temporarily stored by placing it anteriorly towards the nasopharynx or maxillary sinus depending on the surgical area in order not to obscure the surgical field and to protect its vascular supply during surgery. Each step was performed for all of the patients and all surgeries were performed fully endonasal endoscopic.

During each surgical procedure, we used a micro doppler to identify the internal carotid artery. CSF drainage *via* lumbar drainage catheter was applied when dural damage and CSF leakage were detected. We kept the lumbar drainage catheter for 48 hours in order to provide postoperative csf drainage in patients with intraoperative csf leakage. Finally, we went to the IO-MRI unit (3T, MagnetomVida^®^, Siemens Healthineers, Erlangen, Germany) to evaluate the surgical resection rate and the presence of the residual tumor.

### 3-Tesla IO-MRI System

Our 3T MRI site is built next to the operating theatre and end of the operating room corridor that is dedicated to neurosurgical operations. The magnet room interconnects with the operating room corridor by a door RF-shielded. Our system differs from the twin room concept but the contribution of this system to us is that patients in each operating room can go to the IO-MRI unit when the neurosurgeon needs, rather than only accessing the one patient in a single operating room. Also, our 3T IO-MRI unit was designed both to be used intraoperatively and in outpatient clinics. The design of our MR unit gives us the possibility to decrease the cost of the system, as it allows us to use standard surgical equipment.

When the surgeon needs it, the IO-MRI room is properly disinfected and the door of the magnet room and operating room is opened, and the patient is transported to the IO-MRI unit with an MRI compatible stretcher in less than 3 minutes. When the patient is taken to the IO-MRI unit, the anesthesia team monitors the patient and provides the connection with the fixed MRI compatible anesthesia unit in this room. The transport of the patient, the analysis of the images, and the decision-making process take approximately 15 minutes in total. If we decide to continue surgery, the examination procedure is repeated.

### Statistical Methods

For statistical analysis, odds ratio and significance level were calculated with Medcalc software (version 7; Medcalc Software, Mariakerke, Belgium) to evaluate and compare clinical outcome and disease recurrence between groups.

## Results

Between March 2018 and March 2020, 42 patients were operated on for resection of skull base chordomas in our institution and all of them were operated on under IO-MRI. Patients were analyzed retrospectively for the disease recurrence and complications.

Four patients have previous surgery, and none of the patients had received radiation previously. 22 patients were male, and 20 were female (male/female ratio of 1.1:1). The age ranged from 23 to 79 years (mean, 45,6 years). The average follow-up was 22.5 ± 2.4 months (range, 10-36 months). 27 patients presented with headache; 11 with cranial nerve deficit; 2 with hemiparesis; 2 with visual field narrowing. When we examined the reason why most of the patients applied with headache complaints, we found that most of them developed these complaints secondary to underlying causes such as dizziness, drowsiness, blurred vision, nasal obstruction, and nasopharyngeal fullness.

**Figure 1:**
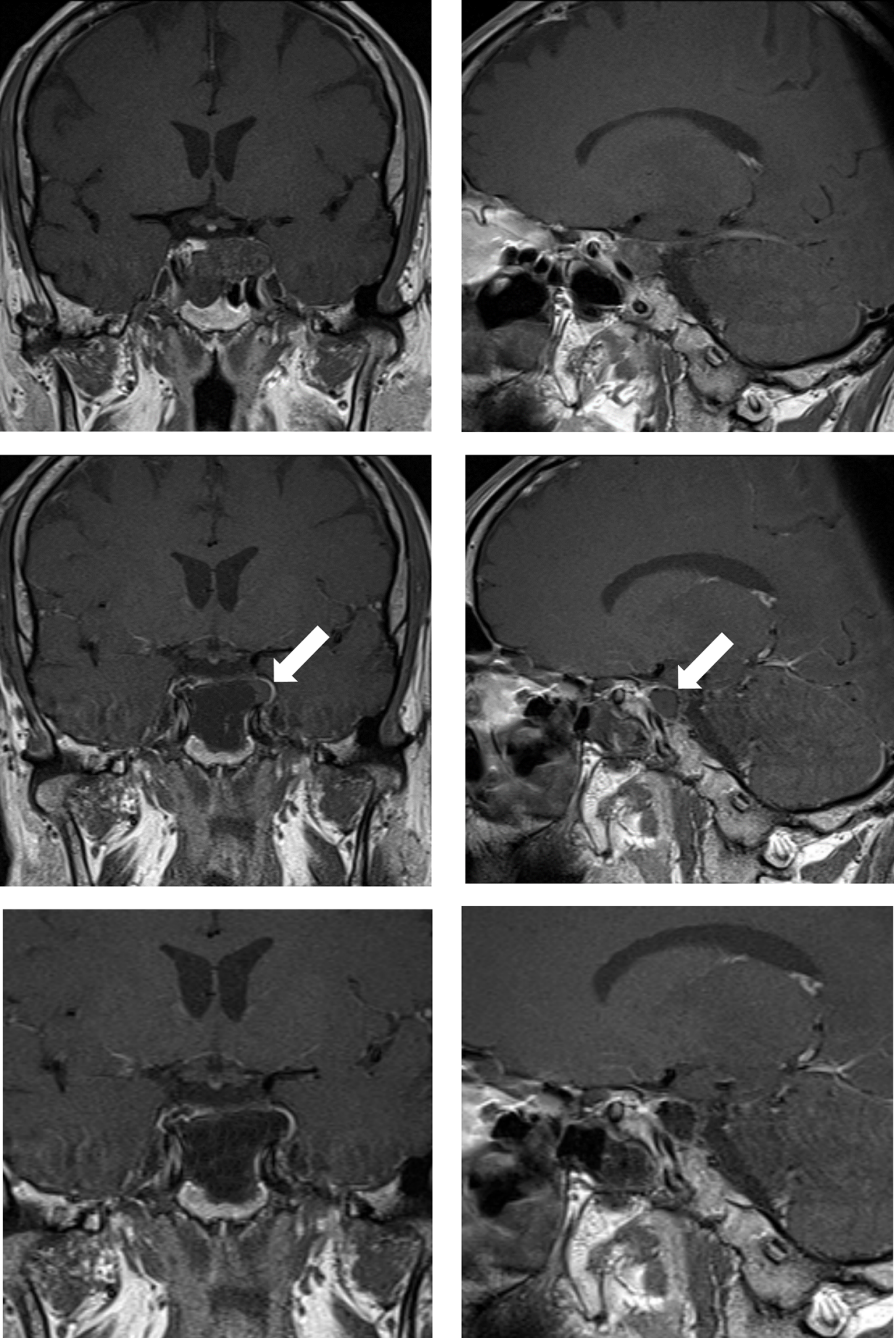
Case sample 1, a mass is detected after the 6th cranial nerve paralysis on the left. Upper row: Preoperative coronal and sagittal contrast-enhanced T1 weighted MRI demonstrates a chordoma that invades the left cavernous sinus and passes lateral to the internal carotid artery. Middle row: Contrast-enhanced T1 weighted IO-MRI slices, the white arrow indicating residual tumor superior to the cavernous sinus. Bottom row: Final contrast-enhanced T1 weighted IO-MRI slices demonstrate gross total surgical resection.

The aim of chordoma surgery is that maximum safe resection of the tumor. Radical surgical resection, defined as no residual tumor with evaluating the final IO-MRI. In 22 patients (52,4%) gross total resection was achieved according to the final IO-MRI. The presence of any residual tumor was defined as subtotal resection and it was achieved in 20 patients (47,6%). In these patients, complete tumor removal was not possible because of extension in the petrous bone (8 patients), pontocerebellar angle (6 patients), prepontine cistern (4 patients), temporobasal (1 patient), cervical axis (1 patient) (**Figure 1**).

**Figure 2:**
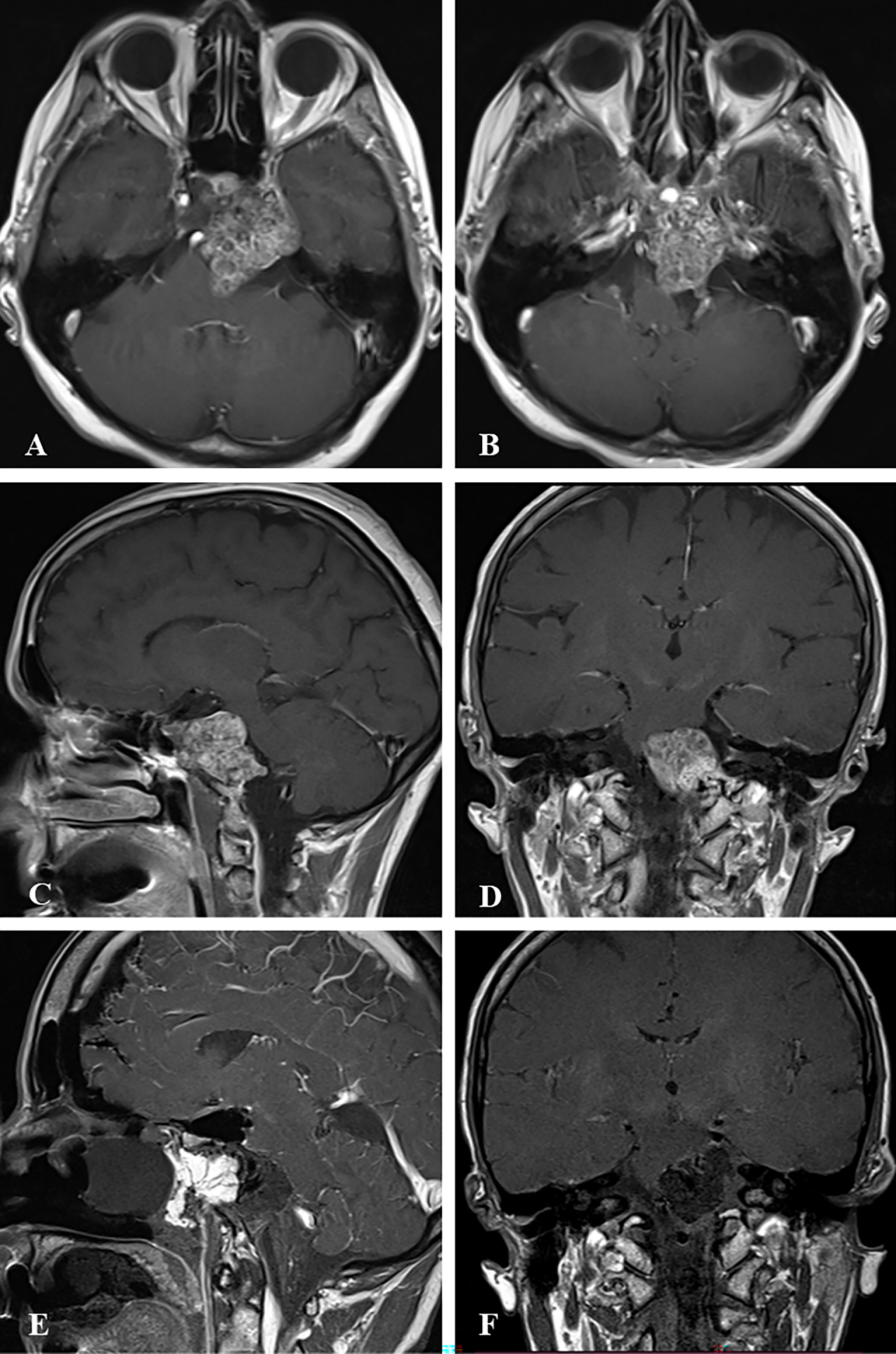
Case sample 2, the patient presenting with headache, diplopia, and reduced sensation of the left side of the face. **(A, B)** Preoperative axial contrast-enhanced T1 weighted MRI shows a tumor invading the left cavernous sinus and compressing the brainstem. **(C)** Preoperative sagittal contrast-enhanced T1 weighted MRI shows a tumor invading the lower clivus and upper cervical spine. **(D)** Preoperative coronal contrast-enhanced T1 weighted MRI shows the tumor relation to the internal acoustic canal and brainstem. **(E)** Final contrast-enhanced T1 weighted IO-MRI demonstrates residual tumor located at the lower clivus and upper cervical spine. This IO-MRI slice also demonstrates the fat graft and nasal package closure. **(F)** Final contrast-enhanced T1 weighted IO-MRI demonstrates tumor resection.

In this case series, the first intraoperative exam demonstrated gross total removal in 15 patients. Other 27 patients demonstrate residual tumor but 14 of these patients’ operations terminated due to achieving the targeted resection rate and proximity of residual lesion with vital neurovascular structures. Residual tumor localizations were prepontine cistern (4 patients), petrous bone (4 patients), pontocerebellar angle (4 patients), temporo basal (1 patient), and cervical axis (1 patient). C1 body and odontoid process invasion were seen in this patient and it was evaluated as cervical axis invasion (**Figure 2**).

**Figure 3:**
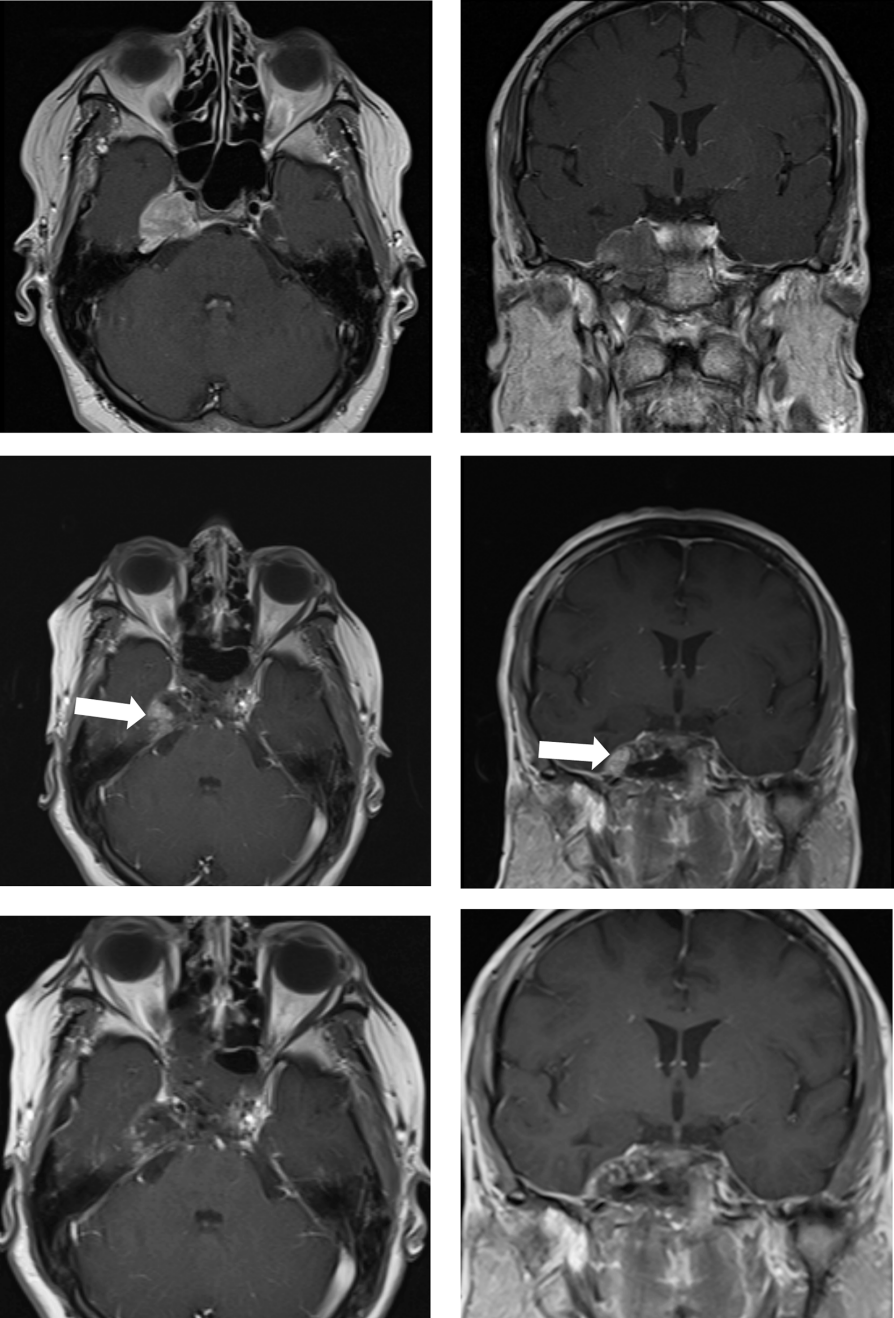
Case sample 3, the patient presenting cranial nerve deficit because of the tumor which invades the right cavernous sinus. Upper row: Preoperative axial and coronal contrast-enhanced T1 weighted MRI shows a tumor invading the right cavernous sinus. Middle row: First contrast-enhanced T1 weighted IO-MRI demonstrates residual tumor located at the inferolateral cavernous sinus. Bottom row: Final contrast-enhanced T1 weighted IO-MRI slices demonstrate gross total surgical resection.

In 13 patients surgical intervention was continued due to the demonstration of resectable remnants which were located in petrous bone (6 patients), cavernous sinus (5 patients), and pontocerebellar angle (2 patients). A second intraoperative exam in 7 of these patients revealed total tumor excision (**Figure 3**). The remnants of these patients were located in cavernous sinus (5 patients) and petrous bone (2 patients). In the remaining 6 patients, the surgery was terminated despite the presence of residual tumor to avoid additional morbidity. These patients’ remnant localization was petrous bone (4 patients) and pontocerebellar angle (2 patients).

**Figure 4:**
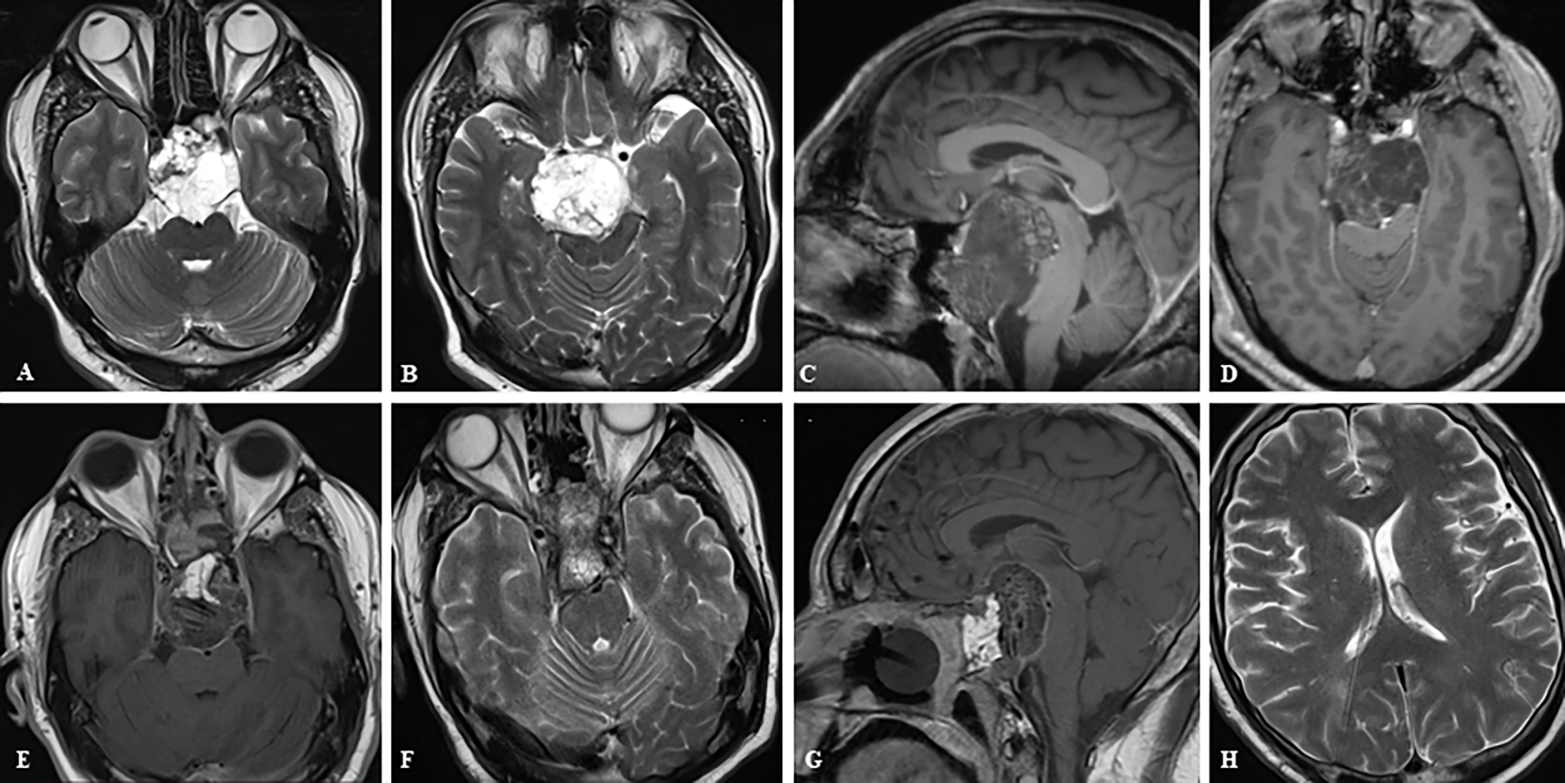
Case sample 4, the patient presenting with headache and impaired cognitive functions. **(A, B)** Preoperative axial T2 weighted MRI shows a huge tumor that extremely compresses the brainstem. **(C, D)** Preoperative sagittal and axial contrast-enhanced T1 weighted MRI shows tumor relation to neurovascular structures. **(E–G)** Final contrast-enhanced T1 weighted IO-MRI shows the gross total tumor resection and also our nasal closure procedure. **(F, G)** The patient developed hydrocephalus in the postoperative period and a ventriculoperitoneal shunt was placed, and these axial T2 weighted MRI images were taken before the patient was discharged after an intensive care period of approximately 3 months **(H)**.

Tumor progression was determined in 2 of the patients who achieved radical surgical resection and 17 of the patients who couldn’t achieve radical surgical resection (**Table 1**). In the postoperative period, 2 patients have developed rhinorrhea and one of them has evolved morbidity after meningitis seconder to the rhinorrhea. Both of the patients who developed rhinorrhea were operated on. And 2 patients underwent ventriculoperitoneal shunt surgery. Communicating hydrocephalus developed in 1 patient in the postoperative period. In other patient, the tumor that filled the prepontine cistern was totally resected, and in the postoperative period, the patient has been developed hydrocephalus, and shunt surgery was applied (**Figure 4**).

**Table 1:** Number of relapses in patients with and without total resection.

	Total resection	Subtotal resection	Total number of patient
Recurrence	2	17	19
Progression free	18	5	23
Total number of patient	20	22	42

Odds ratio 30,6000/z statistic 3,791/Significance level P = 0,0002.

Our findings support today’s knowledge about chordoma; the most important factor for preventing recurrence is total surgical resection. However, what we want to emphasize in this article is the contribution of IO-MRI at this point.

## Discussion

In this study, 42 chordoma patients underwent IO-MRI, 27 patients were found to have residual tumors, and 13 of these patients were evaluated as resectable which was located mostly petrous bone (6 patients), cavernous sinus (5 patients), and pontocerebellar angle (2 patients). Repeated IO-MRI helped achieve gross total resection in 7 of these patients (53.8%) whose tumor was located in the cavernous sinus (5 patients) and petrous bone (2 patients) (**Table 2**).

**Table 2:** Distribution of the patients according to IO-MRI findings.

	IO-MRI/1	IO-MRI/2
Gross total resection	15 patients	7 patients
Subtotal resection (with residue locations)	27 patients *14 patients operation terminated • 4 prepontine cistern • 4 petrous bone • 1 temporobasal • 1 cervical axis *13 patients operation continued • 6 petrous bone • 5 cavernous sinus • 2 pontocerebellar angle	6 patients • 4 petrous bone • 2 pontocerebellar angle

Total tumor resection has been shown to be the most important factor in progression-free survival and overall survival in skull base chordomas (4–8, 16). Controversy over aggressive tumor resection in skull base chordomas arises from the tumor’s relationship to critical neurovascular structures and that total resection often results in significant morbidity (2, 7, 8). Tumor diameter and aggressive growth pattern, previous surgery and radiation therapy have been found to be associated with postoperative morbidity rates (7, 8, 17, 18). Although modern skull base techniques implemented in experienced centers have reduced the mortality and morbidity associated with chordoma resection, chordomas are considered to be tumors that cannot be curable surgically (2, 4–6, 17).

The aim of surgery in tumors that are not suitable for total resection is to decompress critical neurovascular structures, reduce the tumor burden, and establish a pathological diagnosis (4, 6, 11, 16). Thus, adjuvant radiotherapy can be made safer by creating a suitable space between the tumor and radiosensitive structures such as the brain stem and optic nerve (7, 8, 18). In cases of residual tumors or recurrence after surgical treatment, radiation therapy modalities are used in the treatment management, and the radiotherapy response depends on the tumor volume, the radiation dose administered, as well as the biomolecular characteristics of the tumor (2, 7, 8, 10, 11).

Fixed anatomic landmarks play an important role for skull base chordoma surgery because surgeons ensure anatomic orientation and estimation of the degree of resection and the adequacy of neural decompression based on these landmarks (3, 16). However, chordomas tend to destroy surgical landmarks, especially by infiltrating bone structures due to their nature (8, 19). Despite various technical advances, tumor spread and destruction of anatomical landmarks making skull base chordoma surgery are challenging (19–21). In these complex and destructive lesions, direct visual assessment with an operative endoscope may not be sufficient to estimate the residual tumor volume and confirm the completeness of the resection (20, 21). In addition, when large-volume resections are made, it becomes difficult for the surgeon to judge due to the large cavity that occurs (21, 22). At the same time, since the tumor is debulked starting from the center, the reliability of navigation is impaired when the cavity collapses or CSF leakage occurs (4, 9, 14, 22).

IO-MRI is an important tool that can provide real-time feedback on resection grade, residual tumor volume, localization and adequacy of neural decompression (4, 7, 14, 15). The decompression mentioned here is the elimination of tumoral compression, as compression-related changes in neural structures are not expected to disappear immediately in early imaging. Also, IO-MRI can be transferred to the neuronavigation system, making it a useful tool when continuing surgery for residual tumor (15). The main purpose of using IO-MRI in skull base chordoma surgery is to provide a reliable anatomic directive for maximum safe tumor resection with minimum surgical morbidity and mortality (14, 15). Even if IO-MRI does not lead to total resection, it is an important tool to assist the surgeon in preventing neurological comorbidity and making the decision to leave residual tumor (13, 14, 22).

Our study shows that the IO-MRI complements the operative endoscope, thus helping to maximize the degree of resection which is the most important parameter that affects survival.

### Limitations

Since all of our case series consisted of patients operated under IO-MRI, we do not have a control group for skull base chordoma that did not go to IO-MRI. Also, we have limitations in evaluating progression-free survival and tumor recurrence rates since our patient follow-up period is relatively short.

## Conclusions

Our study proves that the use of IO-MRI is a safe method that provides the opportunity to show the degree of resection in skull base chordomas and to evaluate the volume and location of the residual tumor intraoperatively. Hence IO-MRI can improve the life expectancy of patients because it provides an opportunity for both gross total resection and maximal safe resection in cases where total resection is not possible. Also, it makes important contributions in evaluating the adequacy of decompression of neural structures, especially the brainstem which is particularly associated with improvement of clinical status. Another important contribution of IO-MRI is that it enables the evaluation of the relation with residual tumor tissue and radiosensitive neural structures for possible radiation treatments in patients in whom gross total resection is not possible.

## Data Availability Statement

The raw data supporting the conclusions of this article will be made available by the authors, without undue reservation.

## Ethics Statement

This study was reviewed and approved by Clinical Research Ethics Committee at the Gazi University, School of Medicine, Ankara, Turkey. The patients/participants provided their written informed consent to participate in this study.

## Author Contributions

BG, ECe, and ECi contributed to conception and design of the study. ECe organized the database. BG performed the statistical analysis. All surgeries were performed by MS and ECe. BG wrote the first draft of the manuscript. MK, HE, and BK wrote sections of the manuscript. All authors contributed to manuscript revision, read, and approved the submitted version.

## Conflict of Interest

The authors declare that the research was conducted in the absence of any commercial or financial relationships that could be construed as a potential conflict of interest.

## Publisher’s Note

All claims expressed in this article are solely those of the authors and do not necessarily represent those of their affiliated organizations, or those of the publisher, the editors and the reviewers. Any product that may be evaluated in this article, or claim that may be made by its manufacturer, is not guaranteed or endorsed by the publisher.

## Abbreviations

IO-MRI, intraoperative magnetic resonance imaging; MRI, magnetic resonance imaging; CT, computed tomography; CSF, cerebrospinal fluid.
